# Existential Risk and Cost-Effective Biosecurity

**DOI:** 10.1089/hs.2017.0028

**Published:** 2017-08-01

**Authors:** Piers Millett, Andrew Snyder-Beattie

**Keywords:** Biothreat, Catastrophic risk, Existential risk, Cost-effectiveness, Cost-benefit analysis

## Abstract

In the decades to come, advanced bioweapons could threaten human existence. Although the probability of human extinction from bioweapons may be low, the expected value of reducing the risk could still be large, since such risks jeopardize the existence of all future generations. We provide an overview of biotechnological extinction risk, make some rough initial estimates for how severe the risks might be, and compare the cost-effectiveness of reducing these extinction-level risks with existing biosecurity work. We find that reducing human extinction risk can be more cost-effective than reducing smaller-scale risks, even when using conservative estimates. This suggests that the risks are not low enough to ignore and that more ought to be done to prevent the worst-case scenarios.

## Abstract 2

The authors provide an overview of biotechnological extinction risk, make some rough initial estimates for how severe the risks might be, and compare the cost-effectiveness of reducing these extinction-level risks with existing biosecurity work. They find that reducing human extinction risk can be more cost-effective than reducing smaller-scale risks, even when using conservative estimates suggesting that the risks are not low enough to ignore and that more ought to be done to prevent the worst-case scenarios.

How worthwhile is it spending resources to study and mitigate the chance of human extinction from biological risks? The risks of such a catastrophe are presumably low, so a skeptic might argue that addressing such risks would be a waste of scarce resources. In this article, we investigate this position using a cost-effectiveness approach and ultimately conclude that the expected value of reducing these risks is large, especially since such risks jeopardize the existence of all future human lives.

Historically, disease events have been responsible for the greatest death tolls on humanity. The 1918 flu was responsible for more than 50 million deaths,^1^ while smallpox killed perhaps 10 times that many in the 20th century alone.^2^ The Black Death was responsible for killing over 25% of the European population,^3^ while other pandemics, such as the plague of Justinian, are thought to have killed 25 million in the 6th century—constituting over 10% of the world's population at the time.^4^ It is an open question whether a future pandemic could result in outright human extinction or the irreversible collapse of civilization.

A skeptic would have many good reasons to think that existential risk from disease is unlikely. Such a disease would need to spread worldwide to remote populations, overcome rare genetic resistances, and evade detection, cures, and countermeasures. Even evolution itself may work in humanity's favor: Virulence and transmission is often a trade-off, and so evolutionary pressures could push against maximally lethal wild-type pathogens.^5,6^

While these arguments point to a very small risk of human extinction, they do not rule the possibility out entirely. Although rare, there are recorded instances of species going extinct due to disease—primarily in amphibians, but also in 1 mammalian species of rat on Christmas Island.^7,8^ There are also historical examples of large human populations being almost entirely wiped out by disease, especially when multiple diseases were simultaneously introduced into a population without immunity. The most striking examples of total population collapse include native American tribes exposed to European diseases, such as the Massachusett (86% loss of population), Quiripi-Unquachog (95% loss of population), and the Western Abenaki (which suffered a staggering 98% loss of population).^9^

In the modern context, no single disease currently exists that combines the worst-case levels of transmissibility, lethality, resistance to countermeasures, and global reach. But many diseases are proof of principle that each worst-case attribute can be realized independently. For example, some diseases exhibit nearly a 100% case fatality ratio in the absence of treatment, such as rabies or septicemic plague. Other diseases have a track record of spreading to virtually every human community worldwide, such as the 1918 flu,^10^ and seroprevalence studies indicate that other pathogens, such as chickenpox and HSV-1, can successfully reach over 95% of a population.^11,12^ Under optimal virulence theory, natural evolution would be an unlikely source for pathogens with the highest possible levels of transmissibility, virulence, and global reach. But advances in biotechnology might allow the creation of diseases that combine such traits. Recent controversy has already emerged over a number of scientific experiments that resulted in viruses with enhanced transmissibility, lethality, and/or the ability to overcome therapeutics.^13-17^ Other experiments demonstrated that mousepox could be modified to have a 100% case fatality rate and render a vaccine ineffective.^18^ In addition to transmissibility and lethality, studies have shown that other disease traits, such as incubation time, environmental survival, and available vectors, could be modified as well.^19-21^

Although these experiments had scientific merit and were not conducted with malicious intent, their implications are still worrying. This is especially true given that there is also a long historical track record of state-run bioweapon research applying cutting-edge science and technology to design agents not previously seen in nature. The Soviet bioweapons program developed agents with traits such as enhanced virulence, resistance to therapies, greater environmental resilience, increased difficulty to diagnose or treat, and which caused unexpected disease presentations and outcomes.^22^ Delivery capabilities have also been subject to the cutting edge of technical development, with Canadian, US, and UK bioweapon efforts playing a critical role in developing the discipline of aerobiology.^23,24^ While there is no evidence of state-run bioweapons programs directly attempting to develop or deploy bioweapons that would pose an existential risk, the logic of deterrence and mutually assured destruction could create such incentives in more unstable political environments or following a breakdown of the Biological Weapons Convention.^25^ The possibility of a war between great powers could also increase the pressure to use such weapons—during the World Wars, bioweapons were used across multiple continents, with Germany targeting animals in WWI,^26^ and Japan using plague to cause an epidemic in China during WWII.^27^

Non-state actors may also pose a risk, especially those with explicitly omnicidal aims. While rare, there are examples. The Aum Shinrikyo cult in Japan sought biological weapons for the express purpose of causing extinction.^28^ Environmental groups, such as the Gaia Liberation Front, have argued that “we can ensure Gaia's survival only through the extinction of the Humans as a species … we now have the specific technology for doing the job … several different [genetically engineered] viruses could be released”^(quoted in ref. 29)^. Groups such as R.I.S.E. also sought to protect nature by destroying most of humanity with bioweapons.^30^ Fortunately, to date, non-state actors have lacked the capabilities needed to pose a catastrophic bioweapons threat, but this could change in future decades as biotechnology becomes more accessible and the pool of experienced users grows.^31,32^

What is the appropriate response to these speculative extinction threats? A balanced biosecurity portfolio might include investments that reduce a mix of proven and speculative risks, but striking this balance is still difficult given the massive uncertainties around the low-probability, high-consequence risks. In this article, we examine the traditional spectrum of biosecurity risks (ie, biocrimes, bioterrorism, and biowarfare) to categorize biothreats by likelihood and impact, expanding the historical analysis to consider even lower-probability, higher-consequence events (catastrophic risks and existential risks). In order to produce reasoned estimates of the likelihood of different categories of biothreats, we bring together relevant data and theory and produce some first-guess estimates of the likelihood of different categories of biothreat, and we use these initial estimates to compare the cost-effectiveness of reducing existential risks with more traditional biosecurity measures. We emphasize that these models are highly uncertain, and their utility lies more in enabling order-of-magnitude comparisons rather than as a precise measure of the true risk. However, even with the most conservative models, we find that reduction of low-probability, high-consequence risks can be more cost-effective, as measured by quality-adjusted life year per dollar, especially when we account for the lives of future generations. This suggests that despite the low probability of such events, society still ought to invest more in preventing the most extreme possible biosecurity catastrophes.

## The Impact Spectrum of Various Biothreats

**Figure 1.**
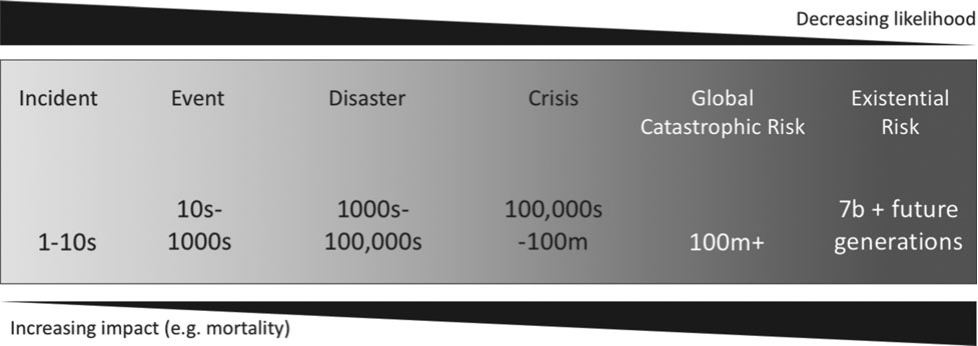
A spectrum of differing impacts and likelihoods from biothreats. Below each category of risk is the number of human fatalities. We loosely define global catastrophic risk as being 100 million fatalities, and existential risk as being the total extinction of humanity. Alternative definitions can be found in previous reports,^33^ as well as within this journal issue.^34^

Here, we use historical data to analyze the probability and severity of biothreats. We place biothreats in 6 loose categories: incidents, events, disasters, crises, global catastrophic risk, and existential risk. Together they form an overlapping spectrum of increasing impact and decreasing likelihood (Figure 1).^*^

The historical use of bioweapons provides useful examples of some categories of biothreats. Biocrimes and bioterrorism provide examples of incidents.^†^ Biological warfare provides examples of events and disasters. These historical examples provide indicative data on likelihood and impact that we can then feed into a cost-effectiveness analysis. We should note that these data are both sparse and sometimes controversial. Where possible, we use multiple datasets to corroborate our numbers, but ultimately the “true rate” of bioweapon attacks is highly uncertain.

### Biocrimes and Bioterrorism

Historically, risks of biocrime^‡^ and bioterrorism^§^ have been limited. A 2015 *Risk and Benefit Analysis for Gain of Function Research* detailed 24 biocrimes between 1990 and 2015 (0.96 per year) and an additional 42 bioterrorism incidents between 1972 and 2014 (1 per year).^36^ This is consistent with other estimates of biocrimes and bioterrorism frequency, which range from 0.35 to 3.5 per year (see supplementary material, part 1, at http://online.liebertpub.com/doi/suppl/10.1089/hs.2017.0028).

Most attacks typically result in no more than a handful of casualties (and many of these events include hoaxes, threats, and attacks that had no casualties at all). For example, the anthrax letter attacks in the United States in 2001, perhaps the most high-profile case in recent years, resulted in only 17 infections with 5 fatalities.^37^ The 2015 *Risk and Benefit Analysis for Gain of Function Research* detailed only a single death from the recorded biocrimes.^**^ Only 1 of the bioterrorism incidents in the report had associated deaths (the 2001 anthrax letter attacks).^36^ Based on this data, for the purposes of this article, we assume that we could expect 1 incident per year resulting in up to tens of deaths.

### Biological Warfare

Academic overviews of biological warfare^††^ detail 7 programs prior to 1945.^38^ A further 9 programs are recorded between 1945 and 1994.^39^ For most of the last century, at least 1 program was active in any given year (Table 1).

**Table 1.** The duration of state-run offensive biological weapons programs detailed in key historical reviews up to 1945 and from 1945 to 2000.^5,6^

*State*	*Duration (Review up to 1945)*	*Duration (Review from 1945-2000)*
Canada	1925-1945	1945-1969
France	1921-1926 and 1935-1940	1947-1972
Germany	1915-1918	—
Hungary	—	1938-1944
Iraq	—	1974-1990
Japan	1931-1945	—
Poland	—	1945-1960?
South Africa	—	1981-1994
Soviet Union	1920-1945	1945-1992
United Kingdom	1925-1945	1945-1957
United States	1942-1945	1945-1969

The actual use of bioweapons by states is less common: Over the 85 years covered by these histories (1915 to 2000), 18 cases of use (or possible use) were recorded, including outbreaks connected to biological warfare (see supplementary material, part 2, at http://online.liebertpub.com/doi/suppl/10.1089/hs.2017.0028). Extrapolating this out (dividing 18 by 85), we would have about a 20% chance per year of biowarfare. It is worth noting the limitations of these data. Most of these events occurred before the introduction of the Biological Weapons Convention and were conducted by countries that no longer have biological weapons programs. Since many of these incidents occurred during infrequent great power wars, we revise our best guess to around 10% chance per year of biowarfare.

We use 2 sets of data to estimate the magnitude of such events. The first dataset was Japanese biological warfare in China,^40^ where records indicate a series of attacks on towns resulted in a mean of 330 casualties per event and 1 case in which an attack resulted in a regional outbreak causing an estimated 30,000 deaths (see supplementary material, part 3, at http://online.liebertpub.com/doi/suppl/10.1089/hs.2017.0028). The second data set came from disease events that were alleged to have an unnatural origin.^41^ In one case study, a point source release of anthrax resulted in at least 66 deaths. In a second case study, a regional epidemic of the same disease resulted in more than 17,000 human cases. While these events were not confirmed as having been caused by biological warfare, contemporary or subsequent analysis has suggested that such an origin was at least feasible. Combined, these figures provide an estimated impact of between 66 to 330 and 17,000 to 30,000.

For the purposes of this analysis, we are assuming the lower boundary figures from biological warfare are indicative of *events*, with a likelihood of 10% per year and an impact ranging between tens and thousands of fatalities. The upper boundary figures from biological warfare are indicative of *disasters*, with a likelihood of 1% per year and an impact range of thousands to tens of thousands of fatalities.^‡‡^

## Global Catastrophic and Existential Risk

Unlike standard biothreats, there is no historical record on which to draw when considering global catastrophic or existential risks. Alternative approaches are required to estimate the likelihood of such an event. Given the high degree of uncertainty, we adopt 3 different approaches to approximate the risk of extinction from bioweapons: utilizing surveys of experts, previous major risk assessments, and simple toy models. These should be taken as initial guesses or rough order-of-magnitude approximations, and not a reliable or precise measure.

### Model 1: Survey of 2008 Global Catastrophic Risk Conference

An informal survey at the 2008 Oxford Global Catastrophic Risk Conference asked participants to estimate the chance that disasters of different types would occur before 2100. Participants had a median risk estimate of 0.05% that a natural pandemic would lead to human extinction by 2100, and a median risk estimate of 2% that an “engineered” pandemic would lead to extinction by 2100.^42^

The advantage of the survey is that it directly measures the quantity that we are interested in: probability of extinction from bioweapons. The disadvantage is that the estimates were likely highly subjective and unreliable, especially as the survey did not account for response bias, and the respondents were not calibrated beforehand. We therefore also turn to other models that, while indirect, provide more objective measures of risk.^§§^

### Model 2: Potentially Pandemic Pathogens

Recent controversial experiments on H5N1 influenza prompted discussions as to the risks of deliberately creating potentially pandemic pathogens. These agents are those that are highly transmissible, capable of uncontrollable spread in human populations, highly virulent, and also possibly able to overcome medical countermeasures.^44^ Previous work in a comprehensive report done by Gryphon Scientific, *Risk and Benefit Analysis of Gain of Function Research,*^36^ has laid out very detailed risk assessments of potentially pandemic pathogen research, suggesting that the annual probability of a global pandemic resulting from an accident with this type of research in the United States is 0.002% to 0.1%. The report also concluded that risks of deliberate misuse were about as serious as the risks of an accidental outbreak, suggesting a 2-fold increase in risk. Assuming that 25% of relevant research is done in the United States as opposed to elsewhere in the world, this gives us a further 4-fold increase in risk. In total, this 8-fold increase in risk gives us a 0.016% to 0.8% chance of a pandemic in the future each year (see supplementary material, part 4, at http://online.liebertpub.com/doi/suppl/10.1089/hs.2017.0028).

The analysis in *Risk and Benefit Analysis of Gain of Function Research* suggested that lab outbreaks from wild-type influenza viruses could result in between 4 million and 80 million deaths,^36^ but others have suggested that if some of the modified pathogens were to escape from a laboratory, they could cause up to 1 billion fatalities.^45^ For the purposes of this model, we assume that for any global pandemic arising from this kind of research, each has only a 1 in 10,000^***^ chance of causing an existential risk. This figure is somewhat arbitrary but serves as an excessively conservative guess that would include worst-case situations in which scientists intentionally cause harm, where civilization permanently collapses following a particularly bad outbreak, or other worst-case scenarios that would result in existential risk. Multiplying the probability of an outbreak with the probability of an existential risk gives us an annual risk probability between 1.6 × 10^–8^ and 8 × 10^–7^.^†††^

### Model 3: Naive Power Law Extrapolation

Previous literature has found that casualty numbers from terrorism and warfare follow a power law distribution, including terrorism from WMDs.^46^ Power laws have the property of being scale invariant, meaning that the ratio in likelihood between events that cause the deaths of 10 people and 10,000 people will be the same as that between 10,000 people and 10,000,000 people.^‡‡‡^ This property results in a distribution with an exceptionally heavy tail, so that the vast majority of events will have very low casualty rates, with a couple of extreme outliers.

Past studies have estimated this ratio for terrorism using biological and chemical weapons to be about 0.5 for 1 order of magnitude,^47^ meaning that an attack that kills 10^x^ people is about 3 times less likely (10^0.5^) than an attack that kills 10^*x*–1^ people (a concrete example is that attacks with more than 1,000 casualties, such as the Aum Shinrikyo attacks, will be about 30 times less probable than an attack that kills a single individual). Extrapolating the power law out, we find that the probability that an attack kills more than 5 billion will be (5 billion)^–0.5^ or 0.000014. Assuming 1 attack per year (extrapolated on the current rate of bio-attacks) and assuming that only 10% of such attacks that kill more than 5 billion eventually lead to extinction (due to the breakdown of society, or other knock-on effects), we get an annual existential risk of 0.0000014 (or 1.4 × 10^–6^).

We can also use similar reasoning for warfare, where we have more reliable data (97 wars between 1820 and 1997, although the data are less specific to biological warfare). The parameter for warfare is 0.41,^47^ suggesting that wars that result in more than 5 billion casualties will comprise (5 billion)^–0.41^ = 0.0001 of all wars. Our estimate assumes that wars will occur with the same frequency as in 1820 to 1997, with 1 new war arising roughly every 2 years. It also assumes that in these extreme outlier scenarios, nuclear or contagious biological weapons would be the cause of such high casualty numbers, and that bioweapons specifically would be responsible for these enormous casualties about 10% of the time (historically bioweapons were deployed in WWI, WWII, and developed but not deployed in the Cold War—constituting a bioweapons threat in every great power war since 1900). Assuming that 10% of biowarfare escalations resulting in more than 5 billion deaths eventually lead to extinction, we get an annual existential risk from biowarfare of 0.0000005 (or 5 × 10^–7^).

Perhaps the most interesting implication of the fatalities following a power law with a small exponent is that the majority of the expected casualties come from rare, catastrophic events. The data also bear this out for warfare and terrorism. The vast majority of US terrorism deaths occurred during 9/11, and the vast majority of terrorism injuries in Japan over the past decades came from a single Aum Shinrikyo attack. Warfare casualties are dominated by the great power wars. This suggests that a typical individual is far more likely to die from a rare, catastrophic attack as opposed to a smaller scale and more common one. If our goal is to reduce the greatest expected number of fatalities, we may be better off devoting resources to preventing the worst possible attacks.

### Why Uncertainty Is Not Cause for Reassurance

Each of our estimates rely to some extent on guesswork and remain highly uncertain. Technological breakthroughs in areas such as diagnostics, vaccines, and therapeutics, as well as vastly improved surveillance, or even eventual space colonization, could reduce the chance of disease-related extinction by many orders of magnitude. Other breakthroughs such as highly distributed DNA synthesis or improved understanding of how to construct and modify diseases could increase or decrease the risks. Destabilizing political forces, the breakdown of the Biological Weapons Convention, or warfare between major world powers could vastly increase the amount of investment in bioweapons and create the incentives to actively use knowledge and biotechnology in destructive ways. Each of these factors suggests that our wide estimates could still be many orders of magnitude off from the true risk in this century. But uncertainty is not cause for reassurance. In instances where the probability of a catastrophe is thought to be extremely low (eg, human extinction from bioweapons), greater uncertainty around the estimates will typically imply greater risk of the catastrophe, as we have reduced confidence that the risk is actually at a low level.^48^^§§§^

Given that our conservative models are based on historical data, they fail to account for the primary source of future risk: technological development that could radically democratize the ability to build advanced bioweapons. If the cost and required expertise of developing bioweapons falls far enough, the world might enter a phase where offensive capabilities dominate defensive ones. Some scholars, such as Martin Rees, think that humanity has about a 50% chance of going extinct due in large part to such technologies.^49^ However, incorporating these intuitions and technological conjectures would mean relying on qualitative arguments that would be far more contentious than our conservative estimates. We therefore proceed to assess the cost-effectiveness on the basis of our conservative models, until superior models of the risk emerge.

### How Bad Would Human Extinction Be?

Human extinction would not only end the 7 billion lives in our current generation, but also cause the loss of all future generations to come. To calculate the humanitarian cost associated with such a catastrophe, one must therefore include the welfare of these future generations. While some have argued that future generations ought to be excluded or discounted when considering ethical actions,^50^ most of the in-depth philosophical work around the topic has concluded that future generations should not be given less inherent value.^51-55^ Therefore, for our calculations, we include future lives in our cost-effectiveness estimate.^****^

The large number of future generations at stake mean that reducing existential risk even by a small amount may have very large expected value. The Earth is thought to be habitable for roughly another billion years;^56^ our closest relative, homo erectus, lasted over 1.6 million years,^57^ and the typical mammalian species also lasts on the order of 1 to 2 million years.^58^ Following Matheny,^29^ if we were to assume that humanity would otherwise maintain a global population of 10 billion for the next 1.6 million years, human extinction would jeopardize on the order of 1.6 × 10^16^ life years.

## Cost-Effective Biosecurity

How should we balance speculative risks of human extinction in a biosecurity portfolio? Here we turn to cost-effectiveness analysis, which is one method of prioritizing public projects.^29^ Cost-effectiveness analysis is helpful if our goal is to maximize the effect of our resources to achieve a measurable aim (such as life-years saved or cases of disease averted). Here we compare the cost-effectiveness of reducing risks in the categories of incidents, events, disasters, and existential risks.

### Calculating Costs

The US federal government was projected to spend almost $13 billion on health security–related programs in 2017.^59^ To our knowledge, there has not been a quantitative assessment of how this spending has reduced the chances of bioterrorism, biowarfare, or even naturally occurring pandemics. However, the World Bank estimates that it would cost $1.9 billion to $3.4 billion per year over 5 years to bring all human and animal health systems up to minimal international standards, and it suggests that these measures would prevent at least 20% of pandemics.^60^^††††^ Many countries do not currently have healthcare systems that meet international standards—for example, in 2014 only 33% of countries reported their national arrangements met those required under the International Health Regulations.^61^

These mitigation measures would be adopted to be effective regardless of whether a disease outbreak originates naturally, accidentally, or deliberately.^‡‡‡‡^ The ability to rapidly detect and characterize the agent involved helps fast-track public health and R&D responses. Acting promptly enables basic public health measures that might decrease the likelihood of spread (such as social distancing) and track its emerging epidemiology (providing critical input for tailoring the responses). Even if we lack existing or candidate vaccines or therapeutics, having the capacity to treat symptoms can have a dramatic impact on case fatality rates.^§§§§^

We therefore assume that strengthening healthcare systems to meet international standards would have an impact on mitigating all types of disease risk, ranging from incidents and events to existential risks.^*****^ We extend the World Bank's assumptions to include bioterrorism and biowarfare—that is, we assume that the healthcare infrastructure would reduce bioterrorism and biowarfare fatalities by 20%. We conservatively assume that existential risks will be reduced by only 1%, since any potential existential risk would likely be deliberately designed to overcome medical countermeasures.

We calculate that purchasing 1 century's worth of global protection in this form would cost on the order of $250 billion, assuming that subsequent maintenance costs are lower but that the entire system needs intermittent upgrading.^†††††^ To calculate the cost per life-year saved, we use the equation C/(N × L × R), where C is the cost of reducing risk, N is the number of biothreats we expect to occur in 1 century, L is the number of life-years lost in such an event, and R is the reduction in risk achieved by spending a given amount (specified by C). For nonextinction risks, we increase L 50 times over to denote 50 life-years saved per life. The denominator N × L × R denotes the total number of life-years saved.^‡‡‡‡‡^ In a subsequent model we also apply a discount rate to represent policymakers concerned only about lives in the short term.

## Results

Including future generations into our cost-effectiveness calculations demonstrates that reducing existential risks, even if they are improbable, can be incredibly cost-effective in expectation (Table 2). Depending on the model used, we estimate that we can purchase 1 quality adjusted life-year in expectation for 10s of dollars (with outliers suggested around 12 cents to $1,600). Even with the most conservative estimates of existential risk, reducing the risk of human extinction is at least 100 times more cost-effective than standard biosecurity interventions, and possibly up to 1 million times more cost-effective.

**Table 2.** Cost-effectiveness estimates of reducing risks of different magnitudes

*Point on Biothreat Spectrum*		*N Expected number of events in 1 century*	*L Expected number of lives lost per event*	*R Reduction in risk by spending $250 billion*	*Cost per life-year saved (assuming 50 years per life)*
Indicative Incident		100	1-10	20%	$25m-$250m
Indicative Event		10	100-1,000	20%	$2.5m-$25m
Indicative Disaster		1	10,000-100,000	20%	$250k-$2.5m
Existential Risk	Model 1	0.0005 to 0.02	10^16^ life years	1%	$0.125-$5.00
	Model 2	1.6 × 10^–6^ to 8 × 10^–5^	10^16^ life years	1%	$31.00-$1,600
	Model 3	5 × 10^–5^ to 1.4 × 10^–4^	10^16^ life years	1%	$18.00-$50.00

It is important to note that this result does not depend on the $250 billion figure—if we found a cheaper intervention that reduced all risks by a similar amount, cost-effectiveness of all the interventions would increase, but the relative merits of reducing existential risk would remain the same.^§§§§§^ There are certainly cheaper ways to reduce the low-level risks of biocrime and bioterrorism, and so our estimates of cost-effectiveness could be far too pessimistic. Examples of cheaper interventions might include dramatically increasing resources for specialized law enforcement prevention and interdiction, or increased surveillance on potential perpetrators. However, there are likely also far cheaper ways of reducing the more extreme risks that threaten extinction, and there is no reason to think similar efficiency gains could not be made in this area as well. Despite the vast resources spent on counterterrorism, governments may have neglected low-probability, high-impact risks.^65,66^ This therefore constitutes a critically underdeveloped area of research, for which there is likely low-hanging fruit.

Even if the humanitarian case for reducing existential risk is clear, most policymakers will be responsible primarily for the interests of a more limited constituency comprising only the current generation and near future.^******^ It is therefore instructive to evaluate how well these cost-effectiveness results hold up when we largely ignore the benefits to future generations. We therefore repeat the cost-effectiveness estimates with a discount rate imposed on the benefits and costs borne in future years, and we find that the merits of reducing existential risk still hold. If we ignore distant future generations by discounting, the benefits of reducing existential risk fall by between 3 and 5 orders of magnitude (with a 1% to 5% discount rate), which is still far more cost-effective than measures to reduce small-scale casualty events. Under our survey model (Model 1), the cost per life-year varies between $1,300 and $52,000 for a 5% discount rate and between $770 and $30,000 for a 1% discount rate. These costs are even competitive with first-world healthcare spending, where typically anything less than $100,000 per quality adjusted life-year is considered a reasonable purchase.^29^

This suggests that even if we are concerned about welfare only in the near term, reducing existential risks from biotechnology is still a cost-effective means of saving expected life if the future chance of an existential risk is anything above 0.0001 per year. Our conservative models (with much lower risk) suggest that existential risk prevention is not cost-effective when compared to basic healthcare spending: Model 2 results in a cost per life-year between $330,000 and $16 million for a 5% discount rate and $190,000 and $9.7 million for a 1% discount rate, while Model 3 results in a cost per life-year of between $190,000 and $500,000 for a 5% discount rate and between $110,000 and $310,000 for a 1% discount rate. These conservative numbers would suggest that healthcare spending is a better purchase than marginal biosecurity funding, but even these numbers still support the notion that we are better off focusing on low-probability, high-impact risks rather than low-casualty biosecurity risks. For a biosecurity portfolio, even policy with limited time horizons is likely better off investing in measures that prevent the worst-case scenarios.

## Conclusions

Although the probability of human extinction from bioweapons may be extremely low, the expected value of reducing the risk (even by a small amount) is still very large, since such risks jeopardize the existence of all future human lives. An initial attempt to estimate the cost-effectiveness of reducing these risks finds that it takes likely between 10 cents and 10s of dollars to save 1 life-year, assuming we value future human lives. Although this result is striking, it is not unprecedented. Similar analysis done by Matheny found that spending $1 billion on an asteroid deflection system would have a similar cost-effectiveness, at about $2.50 per life-year.^29^

Although preventing existential risks might be a far more cost-effective way to save lives than many existing biosecurity measures, this does not imply that we ought to devote all of our resources to protecting against existential risks. Many actions that fall under the rubric of standard health spending also likely reduce existential risk, and many of the resources spent reducing existential risk would in turn help address less extreme risks. Moreover, occasionally there are other opportunities that might be particularly cost-effective—for example, smallpox eradication cost less than $300 million (roughly $1.5 billion in 2017 dollars) and likely saved millions of lives.^68^ The conclusion is thus not that we should abandon all other health interventions for the sake of saving future lives, but rather that on balance we should increase investments that reduce these low-probability, high-stakes risks.

We propose several steps forward. Given the high uncertainty around our estimates, we can expect a high value of information for additional research, implying that resources should be allocated to further assessment of these risks before large sums are directly allocated on the basis of unreliable evidence. Areas for basic research could include examining existential risk using the tools of technological horizon scanning, red-teaming, ecosystem and epidemic modeling, analyzing historical epidemic death tolls, and examining past species that have gone extinct due to disease, among others. And if existential risk could be as important as we claim, more work should be done to assess possible existential risks and countermeasures.

Many actions that would reduce existential risk are already being pursued by those in biosecurity and public health. But there are also measures that would be particularly important in the context of existential risk—including measures that may be unduly neglected without a special focus on existential risk.

One particularly inexpensive measure would be to invest in contingency plans for worst-case scenarios. Countering a pandemic does not typically require a large fraction of worldwide economic output, so there is not a clear path forward for rapidly pivoting to a total war footing in which a large percentage of worldwide GDP is spent on countermeasures. Running small experiments with easily scalable interventions could be a cheap way to explore avenues for rapidly turning resources into protection (examples of such experiments might include paying bounties to individuals or companies to avoid flu infection for a year while conducting essential services, such as power and sanitation).^††††††^

Countering existential risks could also result in reprioritizing current approaches—for example, favoring broad-spectrum diagnostics and countermeasures, as opposed to those tailored to a single pathogen. The worst possible attacks could come from built-up arsenals of multiple pathogens, possibly designed with long incubation periods and traits to overcome vaccination or medical treatment. Platform technologies that allow customizable countermeasures (eg, phages for bacteria, generalized vaccine templates) or pathogen-blind diagnostics (eg, distributed sequencing and improved software to interpret novel pathogens before symptoms occur) will stand a better chance against such threats.^‡‡‡‡‡‡^

An existential risk focus also would place extraordinary weight on avoiding arms races or the widespread weaponization of biotechnology. The near collapse of the 8th Review Conference of the Biological Weapons Convention in December 2016 demonstrates how fragile this regime is and how far current instruments are from the ideal. Strengthening the global norm against biological weapons might go a long way toward reducing the risks associated with state actors. The current 3-person Implementation Support Unit costs less than $1 million per year to support.^71^ In comparison, the 2017 budget for the work of the Organization for the Prohibition of Chemical Weapons is around $77 million (and provides for more than 450 fixed-term posts).^72^ Increasing the human capacity currently focusing on biological weapons risks by several orders of magnitude would be notably cheaper than the costs associated with building core capacities in public and animal health. More generally, any action that reduces the chance of arms races or great power conflict could substantially reduce the probability of existential risk from biotechnology in the century to come.

## Supplementary Material

Supplemental data

## Notes

^*^ While noting that the use of bioweapons can have a wide range of other impacts, including sociopolitical and economic, here we consider their impact purely in terms of fatalities.

^†^ There is considerable uncertainty involved with the dataset on the historical use of biological weapons, including considerable variation in key terms and assumptions, likely knowledge gaps, and motivations for both claiming natural events as unnatural, and vice versa. The numbers used here are intended as indicative and are used to place boundaries on the likelihood and impact of different types of biothreat. As noted elsewhere in this article, the conclusions drawn are considered by orders of magnitude, which helps to address these uncertainties.

^‡^ Biocrimes can be considered to be “the use of a biological agent to kill or make ill a single individual or small group of individuals, motivated by revenge or the desire for monetary gain by extortion, rather than by political, ideological, religious or other beliefs.”^35^

^§^ Bioterrorism can be considered to be “the deliberate release of viruses, bacteria or other agents used to cause illness or death in people, but also in animals or plants. It is aimed at creating casualties, terror, societal disruption, or economic loss, inspired by ideological, religious or political beliefs.”^35^

^**^ A number of other biocrimes involved deliberately infecting another individual with HIV, the results of which were not evident and have not been included in this analysis.

^††^ Biological warfare can be considered to be the “ability to use biological agents in warfare.”^35^

^‡‡^ Whilst there are no documented examples, it is possible that if an attack similar to the one that caused the plague epidemic in China were to be carried out in a modern mega-city, even relatively low infectivity and case fatality rates could result in disasters or even crises. For example, the population of Dhaka, Bangladesh, is approaching 20 million. A disaster would require around 0.5% of its population to die, and a crisis would equate to 5% of the city's population.

^§§^ A more rigorous survey examined the probability of a bioweapons attack in a 10-year timeframe with more than 100 illnesses^43^ and found that opinions varied widely between 1% and 100%, with a mean of 57.5%. While this survey had a superior methodology to the one we cite in model 1, it did not focus on attacks that could result in global catastrophic risk.

^***^ That is to say, if humanity suffered 10,000 pandemics due to potentially pandemic pathogen research, we would expect at least 1 of these pandemics to result in an existential catastrophe.

^†††^ This is the most conservative model because it describes only 1 type of risk that we already know about, so it acts as a lower bound. We need not speculate on future risks from biotechnology to make the case for reducing the risks in this situation.

^‡‡‡^ Specifically, power laws follow the functional form *P* (*x* < *X*) ∝ *x^-a^* where *x* is the number of casualties and *α* is a scaling factor. To calculate the probability of an extreme event (say, an attack that results in over 5 billion deaths), we need an estimate of *α*.

^§§§^ For example, let's say our best guess for a risk is 0.01%, and that we are highly uncertain about this. Even just a 10% chance of underestimating the risk by an order of magnitude will double the risk—with a revised best guess of around 0.02%—while it would take a full 90% chance of overestimating the risk by an order of magnitude to cut the risk in half to around 0.005%. Model uncertainty with respect to low-probability, high-consequence risks is therefore typically additional cause for concern. See Ord et al^48^ for a more in-depth analysis of this problem.

^****^ For a more in-depth discussion on accounting for future generations in cost-effectiveness estimates, we refer the reader to Matheny.^29^

^††††^ Note that the World Bank does not provide any quantitative evidence for this assumption, but we treat it as representing a conservative estimate from experts on how much disease risk would be reduced by a worldwide international health infrastructure.

^‡‡‡‡^ For example, World Health Assembly resolution 58.3, which adopted the revised International Health Regulations in 2005,^62^ made specific reference to “WHA55.16 on global public health response to natural occurrence, accidental release or deliberate use of biological and chemical agents or radionuclear material that affect health,” framing their scope as relevant regardless of the origin of the outbreak.

^§§§§^ Evidence from the recent Ebola public health emergency in West Africa suggested that patients treated in a critical care unit in a developed country were much more likely to survive. The case fatality rate in West Africa was 51%.^63^ The case fatality rate for those treated in Europe and North America was 18.5%.^64^

^*****^ Given the zoonotic nature of many emerging diseases and the recognized importance of adopting a One Health approach when addressing epidemic and pandemic risk, it will be important that both public health and animal health systems are strengthened to meet international standards.

^†††††^ $1.9 billion to $3.4 billion over 5 years is $9.5 billion to $17 billion. If we assume annual maintenance costs are half of those invested ($0.95 billion to $1.7 billion), and a fresh round of reinvestment is needed every 25 years, we get between $114 billion and $204 billion for 100 years of protection. To be even more conservative, we treat this as a lump sum paid up front (we have a discounted model as well). See supplementary material, part 5, at http://online.liebertpub.com/doi/suppl/10.1089/hs.2017.0028 for details.

^‡‡‡‡‡^ We evaluate the first order effects of these interventions and ignore second order spillover effects (such as any economic benefits of innovation that could come with the biosecurity spending). This could be an important oversight, as even short-term and small-scale biosecurity spending could have ramifications for humanity's long-term future (eg, preventing a moderate bioterrorist attack could in turn prevent large wars that escalate or the erosion of norms in civil society, which in turn could evolve into existential risks).

^§§§§§^ Conversely, this ratio will change by altering the expected size of the future population, the lifetime of civilization in the absence of existential risk, or the effectiveness of existential risk reduction measures. There is a simple multiplicative relationship: Cutting the expected value of future civilization in half (by either cutting the population size or the civilization's lifetime in half) will reduce the cost-effectiveness by half, etc. We conduct a simple sensitivity analysis of these parameters in the final supplementary material.

^******^ Cowen and Parfit^67^ describe this justification for discounting the “argument from democracy,” and argue that it typically fails to meet basic criteria to be morally justified (just as a democratic majority in favor of an unjust war would still not be morally justified).

^††††††^ We owe these ideas to Carl Shulman.

^‡‡‡‡‡‡^ While there have been research calls for platform technologies, they have received notably fewer resources than those targeted at more near-term deliverables, such as new vaccines. For example, the USAID call for platform technologies to mitigate future infectious disease threats were looking to fund projects from $100,000 to $1 million,^69^ while the Coalition for Epidemic Preparedness Innovations (CEPI) has received over $500 million to develop vaccines for specific diseases.^70^
