# Supplementary material for: Existential Risk and Cost-Effective Biosecurity
Source: Health Secur. 2017 Aug 1;15(4):373–83. doi: 10.1089/hs.2017.0028 (PMC5576214; doi:10.1089/hs.2017.0028)
Supplement: Supplemental data [file Supp_Data.pdf]

Supplementary Material

1. Biocrime and bioterrorism incidents

Carus[1] reported 153 incidents from 1990 to 1999 but noted only 21 cases of use or attempted use, a mean of 2.1 a year. A background report prepared by the US National Consortium for the Study of Terrorism and Responses to Terrorism reported 74 separate incidents involving biological agents during 1990 to 2011, an average of 3.5 incidents a year.[2] The Rand Database of Worldwide Terrorism Incidents reported 13 biological incidents between 1972 and 2009, resulting in a mean incidence rate of 0.35 a year.[3] Mohtadi and Murshid reported 91 biological weapons incidents between 1950 and 2005, an average of 1.65 per year.[4] There is considerable discrepancy as to what types of incidents are included in these data sets. For example, some include hoaxes and threats; others include incidents that did not result in an actual use, for example, because the perpetrator was interdicted prior to release; a few have attempted to catalogue only when a deliberate disease event has occurred. These differences might have influenced the range of incident rates. Alternatively, the timeframes of the different studies are also divergent, leaving open a possibility that the likelihood of an event has been changing over time.

Finally, we note that there have been a large number of ricin incidents reported in recent years.[5-8] This might suggest that actual levels of biocrimes and bioterrorism events have been underestimated, or it might be the result of recent efforts to develop and implement chemical and biological weapons–specific laws and regulations. In order to avoid overestimating the likelihood of these incidents (and therefore overestimating the cost-effectiveness of measures to mitigate them), as with other assessments of likelihood, we have chosen the most conservative estimates.

2. The impact of unusual disease events alleged to have resulted from state-made bioweapons

Given the paucity of data on the actual impact of biological warfare, to help establish boundaries that are as realistic as possible, we also looked at unusual disease events that have been alleged to have been caused by state-run bioweapons programs. We are not arguing these outbreaks were the result of biological weapons use, but they do provide useful insights into the size, scale, and types of events that national security analysts believed might be caused by biological warfare. As a lower boundary, we considered an unusual outbreak of anthrax in Sverdlovsk in 1979. This was originally reported as having been caused by contaminated meat but was subsequently determined to have been the result of an accidental release of a biological weapons agent from a military facility. The epidemiologic study of this incident suggested that a release of between a few milligrams to almost a gram of agent resulted in at least 66 deaths.* [9] As an upper boundary, we considered a major disease outbreak alleged to have been deliberately initiated by another country: the major anthrax outbreak in southern Africa in 1979.[10] This outbreak resulted in 171,990 cattle and 17,199 human cases.[11]

3. Japanese biological warfare attacks during World War II

Three Japanese attacks during the second World War involved single aeroplanes dropping plague-carrying fleas on grain or other media over Ningbo, Chang The, and Congshan, causing at least 100, 500, and almost 400 deaths (a third of the population), respectively.[12] This provides a mortality rate of 330 per attack. Another attack resulted in a regional outbreak of plague, causing 30,000 deaths in 1947.[13] We note that there is considerable uncertainty with this upper figure.

4. Estimating the number of facilities in the world that might be conducting research on potentially pandemic pathogens

When the US government imposed its funding moratorium on gain-of-function research involving 3 pathogens in November 2014, at least 14 institutions received letters indicating their funding might be affected.[14] To estimate

*In practice, a weapon used for biological warfare may involve the release of a greater quantity of agent, potentially over a denser population, under better environmental conditions. This is probably an underestimation of the impact of a biological warfare attack.

the total number of facilities in the world, we assume that there are similar numbers of research institutes undertaking research on potentially pandemic pathogens in China, the European Union, and the rest of the world, providing a total of approximately 56 laboratories.

5. Estimating the cost of improving human and animal health systems to meet international standards

The World Bank has developed a cost estimate of \$1.9 billion to \$3.4 billion per year over 5 years to bring all human and animal health systems up to minimal international standards.[15] This comes to a total cost of between \$9.5 billion and \$17 billion. These costs were bounded by 2 separate estimates of the likelihood of a crossover event (in which an animal pathogen evolves to spread in humans and causes a significant outbreak in an immunologically naive population) and the relative ratio of human and animal disease events (as dealing with human disease is more expensive than dealing with animal disease). One estimate was developed by the World Bank, and the other was put together by an external panel of experts.

These figures do not include costs for longer-term maintenance of such a capability. Presumably, for several years the maintenance costs should be less than the mean annual costs for building the necessary capacity. For the purposes of this estimate, we are assuming the maintenance costs are approximately half those of the initial investment, or between \$0.95 billion and \$1.7 billion per year.

We are also assuming that both wear and tear on the system, as well as improvements in the underlying technologies, will necessitate further capital assessment to renew the system at regular points. We are assuming that this will be necessary about every 25 years (with a 5-year window for capital investment and a further 20 years of maintenance). Based on these assumptions, building and maintaining a global animal and public health infrastructure that meets international standards for the next century would be between \$114 billion and \$204 billion. As a result, we are estimating that the cost of mitigating biothreats as \$250 billion.

6. Value of future human civilization: a sensitivity analysis

Following the analysis done in (27), we assumed that a future human civilization would have 10^16 life-years—a population of 10 billion living for 1 million years, for example (although it could also represent a population of only 1 billion living through Earth's lifetime of 1 billion years). Some may argue that human civilization is unlikely to be that long-lasting, while others would argue that if we avoided existential risk, there could be more than 10^30 humans living in the future.[16] We also assumed that spending \$250 billion would only reduce the risks by 1%. How sensitive are our results to changing these assumptions?

The relationship is a simple multiplicative one—cutting the value of civilization in half (either by reducing its population or its lifetime), or reducing the risk by half with the same amount of money, will reduce the cost-effectiveness by half as well. We have some graphs to demonstrate this (plotted on a log-log scale to enable wide exploration of parameters). We also show a graph of the impact that discounting has on the cost-effectiveness analysis.

The cost-effectiveness of existential risk reduction is proportional to the expected size of future civilization. In this log-log plot, we compare the cost-effectiveness of reducing biological existential risk with the size of a human population that lasts for 1 million years. If we assume future human population will be diminished to only 1 million individuals for the remainder of humanity's lifetime, cost-effectiveness dramatically decreases to between \$10,000 and \$100,000,000 per life-year. Conversely, if we think that humanity will go on to expand beyond Earth and eventually reach an enormous population of 1,000 trillion, the cost-effectiveness becomes extraordinary, with less than one-tenth of a cent purchasing a life-year.

The cost-effectiveness of existential risk reduction is proportional to the expected reduction of existential risk (in absolute percentage terms, not relative terms). If the intervention reduces existential risk by 10% rather than 1%, it will be 10 times cheaper to save a life-year.

If we assume that future generations are not included in our calculations by applying a discount rate, we find that cost-effectiveness ranges between \$1,000 and \$10,000,000 per life-year. This result is not very sensitive to the discount rate, but more sensitive to whether we decide to discount at all (undiscounted cost-effectiveness ranges between about 10 cents and \$1,600).

References

1. Carus WS. Bioterrorism and biocrimes: the illicit use of biological agents since 1900. Working paper. Washington, DC: National Defense University; 2001. https://fas.org/irp/threat/cbw/carus.pdf. Accessed July 21, 2017.
2. National Consortium for the Study of Terrorism and Responses to Terrorism. Ricin Letters Mailed to President and Senator. START Background Report. 2013. https://www.start.umd.edu/sites/default/files/files/publications/br/STARTBackgroundReport_RicinMailings_April2013.pdf. Accessed July 21, 2017.
3. RAND National Memorial Institute for the Prevention of Terrorism. About the RAND database of worldwide terrorism incidents. https://www.rand.org/nsrd/projects/terrorism-incidents/about.html. Accessed July 21, 2017.
4. Mohtadi H, Murshid A. A global chronology of incidents of chemical, biological, radioactive and nuclear attacks: 1950-2005. National Centre for Food Protection and Defense. Diss. Department of Homeland Security; July 2006.
5. Halliday J. Man ordered 'Breaking Bad-style' ricin delivery from FBI agent, court hears. *Guardian* July 21, 2015. https://www.theguardian.com/uk-news/2015/jul/21/breaking-bad-style-ricin-delivery-dark-web-mohammed-ammer-ali-trial. Accessed July 21, 2017.
6. BBC News. Abrin: Boy, 16, sentenced after ordering deadly toxin online. *BBC* April 20, 2015. http://www.bbc.com/news/uk-england-manchester-32383135. Accessed July 21, 2017.

7. US Department of Justice. Florida man sentenced to 110 months in prison for conspiring to murder a foreign national and for sale and smuggling of deadly toxins. February 18, 2015. https://www.fbi.gov/contact-us/field-offices/newark/news/press-releases/florida-man-sentenced-to-110-months-in-prison-for-conspiring-to-murder-a-foreign-national-and-for-sale-and-smuggling-of-deadly-toxins. Accessed June 30, 2017.
8. Johnson EM. Ex-New Jersey pharmacist gets more than six years in prison for trying to make ricin. January 30, 2015. http://www.reuters.com/article/us-usa-new-jersey-ricin-idUSKBN0L32JA20150130. Accessed June 30, 2017.
9. Meselson M, Guillemin J, Hugh-Jones M, et al. The Sverdlovsk anthrax outbreak of 1979. *Science* 1994;266(5188):1202.
10. Martinez I. The history of the use of bacteriological and chemical agents during Zimbabwe's liberation war of 1965-80 by Rhodesian forces. *Third World Quarterly* 2002;23(6): 1159-1179.
11. Wilson JM, Brediger W, Albright TP, et al. Reanalysis of the anthrax epidemic in Rhodesia, 1978-1984. *PeerJ* 2016;4:e2686.
12. Beam TE, Sparacino LR. *Military Medical Ethics (Volume 2).* Washington, DC: Office of The Surgeon General at TMM Publications, Borden Institute, Walter Reed Army Medical Center; 2003:382-383.
13. Frischknecht F. The history of biological warfare. *EMBO Rep* 2003;4(6S):S47-S52.
14. Kaiser J. Moratorium on risky virology studies leaves work at 14 institutions in limbo. *ScienceInsider* 2014;5(6):e02366-14.
15. World Bank. *People, Pathogens and Our Planet, Volume 2: The Economics of One Health.* Washington, DC: World Bank; 2012. http://www.iucn-whsg.org/sites/default/files/People,%20Pathogens%20and%20Our%20Planet.pdf. Accessed July 20, 2017.
16. Bostrom N. Astronomical waste: the opportunity cost of delayed technological development. *Utilitas* 2003;15(3):308-314.

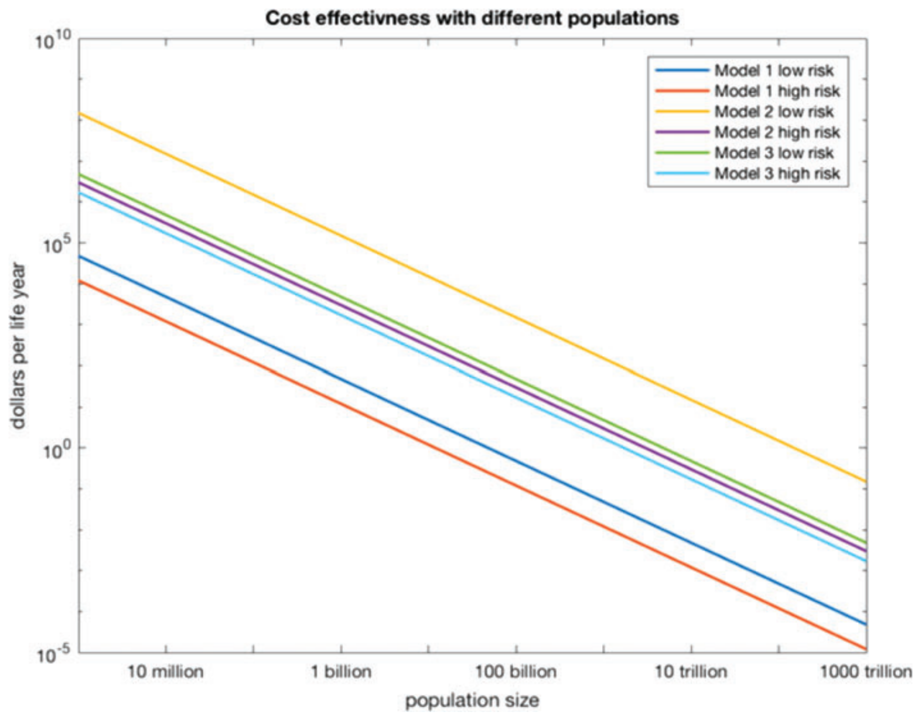

Supplementary Figure S1. Cost-effectiveness with differing population sizes over 1 million years

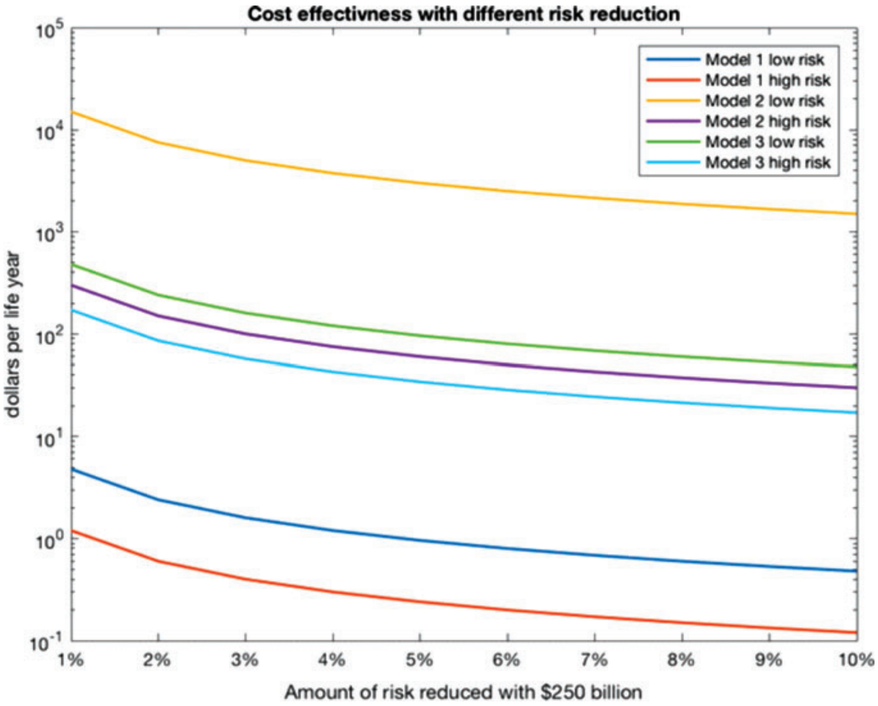

Supplementary Figure S2. Cost-effectiveness with differing effectiveness of intervention

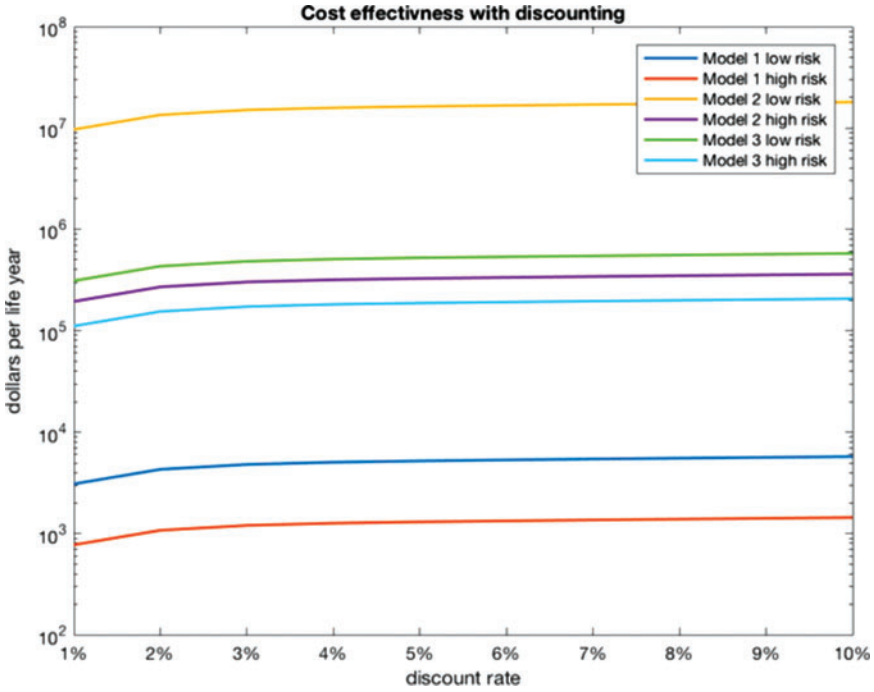

Supplementary Figure S3. Cost-effectiveness with differing discount rates
